# Advice upon Medical Subjects

**Published:** 1869-07

**Authors:** 


### Domestic Medicine.

### Advice Upon Medical Subjects.

BY THE EDITOR.

WORM TEA FOR CHILDREN.—If you have reason to suspect worms in your children prepare the following tea and give about one gill three times daily, before meals, until the bowels are freely moved:

Take of the leaves of senna, ½ oz; pink root ½ oz; bruise and pour on boiling water one pint; steep without boiling. Sweeten well, and add half as much milk. When cool enough, a child from five to eight years old may drink as above directed.

---

BURNS AND SCALDS.—If housekeepers will always keep a small phial of the oil of cloves, well corked, among their family medicines, they will find it of great value as an application to burns and scalds, which are so liable to occur in every household. The oil should be applied by means of a feather, as soon as the injury is received, as it will usually prevent any blister forming, and will always give relief from pain. The oil should be renewed once a year, in order to have it effective.

---

DISEASED GUMS.—We frequently hear young folks complain of "sore gums" and an inability to clean their teeth in consequence of the pain produced by the use of the brush.

This soreness can be removed by observing the following directions: Go to your druggist and have him prepare you the following lotion:—Carbolic acid, one scruple; rectified spirits of wine, two drachms; distilled water, six ounces. Mix. Apply this to your gums and teeth morning and evening, by means of a moderately stiff brush. The first two or three applications may be painful, but the soreness will rapidly subside under this treatment.

---

HABITUAL CONSTIPATION.—Should the constipation be very obstinate, the following formula can be prepared by any competent druggist, and one pill taken night and morning, before eating.

℞.

Ext. rhei alcoholici, ℥ ss.
Ext. taraxaci, gr. xxiv.
Quinæ Sulphatis, gr. ij.

Fiat mass., in pilulas xij div.

A correspondent has complained that such formulæ as the above cannot be read by the people generally. We have only to say, in reply, that they are not intended to be compounded by everybody, but by a competent druggist only. Exhibit all formulæ under this head to any druggist who understands his business and he will at once supply you with the needed remedy.

---

COUP DE SOLEIL, OR SUN STROKE.—Frequently lives are lost from the effects of sun-stroke, that might have been saved had the bystanders acted intelligently and promptly in such cases. Never attempt to remove a man to his home who has fallen from sunstroke—by so doing you waste much valuable time that should be devoted to his treatment. Simply carry him to the shade of some tree or fence, and allow him to lay upon his back on the ground, raising his head and shoulders slightly by folding his coat and placing it under him. Open his shirt and expose his chest to the air—rub his breast, arms, and legs in alcohol or whisky, and dash cold water upon his head. Procure ice as speedily as possible, pound it fine, place it in a salt sack or between the layers of a towel, and apply it to his head. Place another layer of pounded ice to the nape of the neck, meanwhile rubbing his breast and extremities with the whisky. As soon as he can swallow, give him a little brandy and water. You will soon restore him to consciousness, when he can be removed to his residence and placed under the care of his physician.

---

COLIC OF INFANTS.—Babies are continually liable to attacks of severe colic, when mothers usually ply them with laudanum, or what is the same thing, the "soothing syrups" kept in the shops. By the administration of such nostrums the child's digestive apparatus is enfeebled—its bowels constipated and the foundation for a

ARMY MEDICAL LIBRARY 14595 WASHINGTON D. C. U. S. A.

more serious disease is laid. The following treatment will be far more efficacious, and will leave no such train of evils in its wake.

Place the child in a warm bath—relieve the bowels by an injection of warm water—give it a few drops of brandy, or sal volatile, in some warm milk. After eight minutes remove it from the bath, carefully dry it, and apply a hot flaxseed poultice, on which ten or fifteen drops of laudanum has been dropped, to its abdomen. Wrap the child in hot flannels and lay it away in its cradle—it will soon fall into a quiet sleep.

This may be more troublesome than to administer laudanum or "soothing syrup," but it will be, by far, the best treatment for the little one, and you will soon have the satisfaction of seeing its colicy symptoms disappear entirely.

---

DIARRHŒA OF CHILDREN.—Now that hot weather is upon us, we will find our little ones suffering from "bowel complaint," which should be speedily checked or very serious consequences are liable to ensue.

"As an "ounce of preventative is worth a pound of cure," see to it that your children are not allowed to cram their little bellies with cherries, berries, and other fruits. A moderate indulgence in such luxuries will do good, but our little folks would eat of articles of this character incessantly, were they allowed so to do.

If you suspect the diarrhœa is caused by indulgence in fruit, either ripe or unripe, the following mixture may be prepared at your drug store, and given according to direction :—

R. Tinct. Opii. ♏ viij.

Ol. Ricini ʒj.

Syrup. Zingib.,

Mucilag. Acaciæ ā ā ℥ j.

M.

A teaspoonful may be given three times per. day, to a child from 3 to 6 years old.

---

SUMMER BATHING.—Now that the season for bathing has arrived, we should be careful not to abuse the privilege extended to us. Bathing, in a clear running stream, after sundown, is as delightful as it is refreshing and healthful. In walking to the bathing-place, be careful to go leisurely, and, if upon arriving at the stream, you should feel uncomfortably warm from your exertion, rest awhile, until your circulation becomes tranquil. Never remain in the water more than twenty minutes, and provide yourself with a large towel with which dry your skin thoroughly. A bath should never be taken immediately after eating—after a brisk walk, or during the heat of the day.

Boys *will* "swim"—they take to the water as naturally as ducks—therefore forbid them not, but go with your sons, in the evening and allow them twenty minutes play in the water—they will enjoy it all the more to have father with them, and will then, most likely, not disobey his orders not to swim in the middle of the day.

---

FALLING OUT OF HAIR.—In another column we have warned our readers against the use of certain "hair-restorers," so that it is proper that we should now give them a safe and rational treatment for the diseased scalp :

First remove all dandruff from the scalp with the following mixture :

Purified carbonate of Potash, 1 oz.
Rain water, 1 qt.
Mix.

Apply a sufficient quantity to the scalp to moisten it thoroughly, then rub it well with the hands, or a stiff brush, after which rinse out the hair in soft water, drying it well with a coarse towel.

Next have the following "Hair Invigorator" made and use it daily as a dressing. It will prevent the hair from falling out, impart new vigor to its growth, and will be found far superior to the patent nostrums of the day, besides costing much less.

Take Bay Rum, 1 pt.

Alcohol, ½ pt.

Caster Oil, 1 oz.

Carbonate of Amonia, ¼ oz.

Tincture of Cantharides, ½ oz.

Mix, and shake well before using.

---

PILES.—This distressing and very prevalent trouble of the bowel can frequently be cured and always relieved by observing the following course of treatment :

First, it is absolutely necessary that the bowels should be kept unobstructed, and for such persons who are habitually constipated, it will be well for them to have the following pills prepared, one of which should be taken every second night, until the difficulty is overcome :

Take Podophylin, 5 gr.
Extract Butternut, 8 gr.
Capsicum, 6 gr.
Sulphur, 5 gr.

Mix, and make fifteen pills.

Next obtain of glycerine two ounces, in which place twenty grains of tannin, when thoroughly dissolved, apply the mixture to the piles morning and evening. By following up this treatment for one month, you will most likely suceeed in curing the difficulty.

---

CHOLERA MIXTURE.—During warm weather every family should be prepared with a remedy with which to combat cholera. The disease attacks one without any previous warning, and urgent and expeditious measures are always necessary in handling it. Have the following mixture made at a drug store and always keep it on hand, viz:

Laudanum,
Tincture Camphor,
Tincture Capsicum, of each two (2) drachms. Mix, and give a half teaspoonful three times per day, if the symptoms are not severe. The same dose may be given as often as every hour in the worst forms of the disease, and mustard plasters should be applied to the soles of the feet, palms of the hands, back of the neck and over the abdomen. The abdomen should be rubbed with some stimulating application, as tincture of capsicum or camphor. While your are pursuing this treatment send for a physician, under whose eye and direction the balance of the treatment should be conducted.

The above mixture will be found useful—in smaller doses—in the "bowel complaints" to which children are subject at this season of the year. Give them from 5 to 20 drops on sugar, three times per day.

